# Shot-noise limited, supercontinuum-based optical coherence tomography

**DOI:** 10.1038/s41377-021-00574-x

**Published:** 2021-06-28

**Authors:** Shreesha Rao D. S., Mikkel Jensen, Lars Grüner-Nielsen, Jesper Toft Olsen, Peter Heiduschka, Björn Kemper, Jürgen Schnekenburger, Martin Glud, Mette Mogensen, Niels Møller Israelsen, Ole Bang

**Affiliations:** 1 DTU Fotonik, Dept. of Photonics Engineering, Technical University of Denmark, Ørsteds Plads, 2800 Kongens Lyngby, Denmark; 2 NKT Photonics A/S, Blokken 84, 3460 Birkerød, Denmark; 3 Department of Ophthalmology, University of Münster Medical Centre, Domagkstr. 15, D-48149 Münster, Germany; 4 Biomedical Technology Center of the Medical Faculty, University of Münster, Mendelstr. 17, D-48149 Münster, Germany; 5 Department of Dermatology, Bisbebjerg Hospital, University of Copenhagen, Bispebjerg Bakke 23, 2400 Copenhagen NV, Denmark

**Keywords:** Supercontinuum generation, Biophotonics, Fibre lasers

## Abstract

We present the first demonstration of shot-noise limited supercontinuum-based spectral domain optical coherence tomography (SD-OCT) with an axial resolution of 5.9 μm at a center wavelength of 1370 nm. Current supercontinuum-based SD-OCT systems cannot be operated in the shot-noise limited detection regime because of severe pulse-to-pulse relative intensity noise of the supercontinuum source. To overcome this disadvantage, we have developed a low-noise supercontinuum source based on an all-normal dispersion (ANDi) fiber, pumped by a femtosecond laser. The noise performance of our 90 MHz ANDi fiber-based supercontinuum source is compared to that of two commercial sources operating at 80 and 320 MHz repetition rate. We show that the low-noise of the ANDi fiber-based supercontinuum source improves the OCT images significantly in terms of both higher contrast, better sensitivity, and improved penetration. From SD-OCT imaging of skin, retina, and multilayer stacks we conclude that supercontinuum-based SD-OCT can enter the domain of shot-noise limited detection.

## Graphical abstract

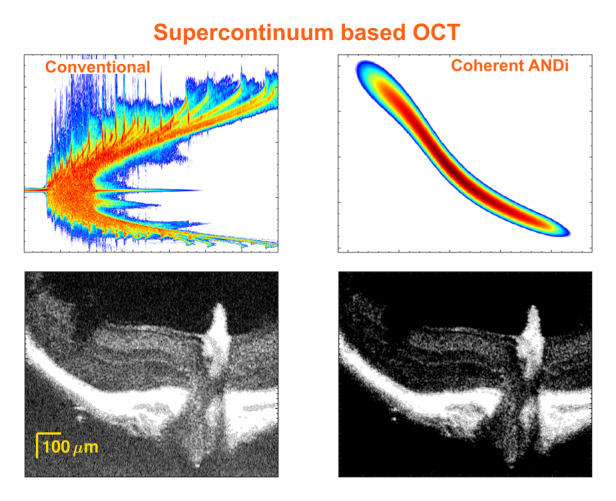

## Introduction

Optical coherence tomography (OCT) relies on white light interferometry to noninvasively image translucent samples^1^. Since its invention three decades ago, OCT has undergone an exceptional technological development manifested in imaging speeds increased by more than a factor of a million^2^, sensitivities improved by more than 16 dB^3^, and the resolution enhanced to single cell levels^4^. These improvements in speed and sensitivity are largely down to the shift from the original time-domain (TD) OCT to Fourier domain (FD) OCT^5^. In TD-OCT, a single photodiode detects the interferometric signal while a mirror is scanning the reference arm of the interferometer. In FD-OCT however, the spectral components of the white light source are detected independently, either with a spectrometer [termed spectral domain (SD) OCT] or with a wavelength-swept laser and a fast photodiode [termed swept source (SS) OCT]. A Fourier transform builds an axial scan (A-scan) similar to that created by scanning in TD-OCT. The advantage of both FD methods over TD-OCT is twofold: firstly, eliminating the need for a scanning mirror greatly increases detection speed. Secondly, the independent detection of the spectral components improves the sensitivity^6–9^. This technological advancement has facilitated the widespread clinical use of high-resolution OCT in ophthalmology^10^ as well as the extensive and very active research in other medical fields, such as dermatology^11^, oncology^12^, and gastroenterology^13^.

Since OCT relies on white light interferometry, the axial resolution (Δz) of an OCT system is equal to the coherence length of the employed light source. The coherence length, *l*_*c*_, scales proportionally with the square of the center wavelength of the light source, *λ*_0_, and inversely proportional with the bandwidth, Δ*λ*. For a light source with a Gaussian spectrum, we have ${l}_{c}\,=\,{{\Delta }}z\,=\,(2{\mathrm{ln}}\,2/\pi )\,\times\, ({\lambda }_{0}^{2}/{{\Delta }}\lambda )\,\times\, (1/n)$, where *n* is the refractive index of the sample. Reducing the center wavelength to improve the resolution affects both the absorption and scattering of the light in the sample and may therefore reduce the penetration. A more controllable approach to improve the resolution is thus to choose a light source with a wide bandwidth.

**Fig. 1:**
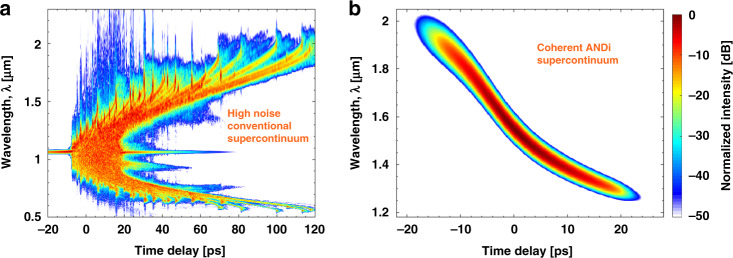
Output of SC sources. Simulated single-shot output spectrograms of (**a**) a typical conventional long-pulse pumped SC source and (**b**) a typical fully coherent ANDi SC source

Emerging commercially over the last 15 years, supercontinuum (SC) sources have had an instant impact in the life sciences. An SC-based confocal microscope was for example voted as a top ten innovation in 2008^14^. SC sources have also been an increasingly popular choice in OCT when ultra-high axial resolution is the key metric^11,15^. This is due to their multi-octave spanning spectra, small footprints, robustness, and long-term stability. However, the main disadvantage of SC laser sources in OCT is their severe intensity noise/pulse-to-pulse relative intensity fluctuations, which limits the sensitivity of OCT systems due to a noise level far from the ideal shot-noise limit^16^. Current state-of the art SC-based SD-OCT systems use high (320 MHz) repetition rate SC sources to average out some of the intensity noise^17,18^. The intensity noise is common for all conventional SC sources, and the reason is found in the underlying physics: In most commercial SC sources, the broadband light is generated by launching a long [picosecond (ps) or nanosecond (ns)] light pulse through a nonlinear optical fiber. Complex interplay between linear and nonlinear effects create solitons and dispersive waves (DWs) that together constitute an ultrawide spectrum covering several octaves, as seen in the typical output spectrogram shown in Fig. 1a. In the regime of long pulses, the initial broadening of the SC is generated by nonlinear amplification of quantum noise^19^, and the resulting spectra are thus particularly noisy and uncorrelated from pulse-to-pulse. The large integration time needed to reduce the effect of intensity noise to an acceptable level when using conventional SC sources, makes the OCT system unsuitable to image moving objects, such as a flickering eye in ophthalmology. Hence, there is a pressing need for SC sources without any excess noise. The short pulse [femtosecond (fs)] regime has long promised ultra-low-noise SC from coherent spectral broadening^20–22^ (a typical output spectrogram is shown in Fig. 1b). However, polarization coupling^23^, nonlinear effects^24^, and pump laser noise^25^ introduce severe restrictions on the combination of pulse peak power, pulse length, and fiber length that guarantees low-noise behavior. This limitation makes short-pulsed SC sources less broadband and of lower power than their long-pulsed counterparts. As a result, fs-based SC sources are only just emerging in the commercial market. Even research systems have hardly been used in OCT, with the exception of Kawagoe et al.^26^ who demonstrated SD-OCT with an SC source utilizing short pump pulses. While demonstrating imaging of a mouse brain with good sensitivity, Kawagoe et al. did not quantify the noise performance of the SC source or analyze the performance in OCT imaging, and thus it has yet to be demonstrated that shot-noise limited OCT can be achieved with an SC source.

To answer this fundamental question and for the first time demonstrate that shot-noise limited SC-based SD-OCT is indeed possible, we have developed an SD-OCT system operating around 1370 nm, which uses a low-noise SC source that we have designed in-house specifically for this application. The low-noise SC source uses a fiber with weak all-normal dispersion (ANDi) pumped by a fs laser, in which case the spectral broadening is dominated by the coherent processes of self-phase modulation (SPM) and optical wave-breaking (OWB)^27,28^. We ensured that the normal dispersion is weak and the pulse length is short, in order to specifically avoid noise-generating effects, such as parametric Raman noise^24^ and polarization mode instability^23^.

We first compare, one-to-one, the noise characteristics of the OCT system using both commercially available SC sources, SuperK extreme sources operating at 80 and 320 MHz (from NKT Photonics), as well as our ANDi fiber-based low-noise SC source operating at 90 MHz, from now on referred to as just the ANDi SC source. The 320 MHz SuperK extreme source represents today’s state-of-the-art in SC sources for SD-OCT due to its high repetition rate. We use all three sources to image a tape-layer phantom, healthy skin, an ex vivo mouse retina, and ex vivo fat tissue and compare the images. We demonstrate that in all cases the SD-OCT system with the ANDi fiber-based low-noise SC source provides superior and shot-noise limited performance.

## Results

### Noise characteristics

**Fig. 2:**
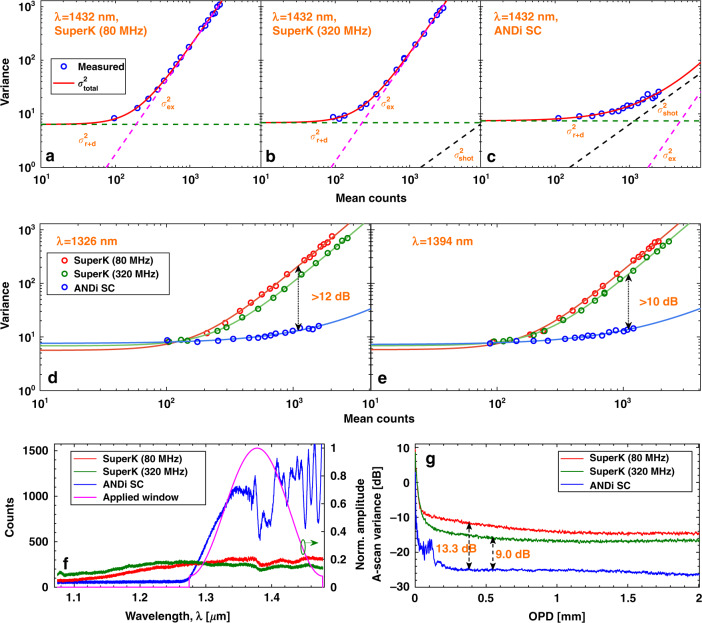
OCT system noise characterization. The variance in counts versus the mean counts (over 1024 measurements) at 1432 nm for the 80 MHz SuperK extreme (**a**), 320 MHz SuperK extreme (**b**), and ANDi SC source with 90 MHz repetition rate (**c**). Comparison of the variance in counts versus the mean counts for the 80 MHz SuperK extreme, 320 MHz SuperK extreme, and ANDi SC source with 90 MHz repetition rate at 1326 nm (**d**), and 1394 nm (**e**). Spectral counts across the spectrometer bandwidth for the three sources, when the counts at 1432 nm is fixed at the optimum value for imaging (250 counts for SuperK extreme sources and 1000 counts for the ANDi SC source) (**f**). The magenta curve shows the window used in the signal processing. **g** Corresponding variance in A-scans over 1024 measurements. In (**d**–**g**) the 80 MHz SuperK extreme, 320 MHz SuperK extreme, and ANDi SC source are shown as red, green, and blue curves, respectively. All measurements are taken with blocked sample arm

The main unit of our SD-OCT setup is a Michelson interferometer (see Fig. 6a in the Materials and methods section for further details). Noise characterization of the OCT system was done with each of the three sources in the wavelength range 1280–1478 nm, with identical spectrometer operation. The sample arm was blocked and the photon-counts measured in the spectrometer were recorded for various reference arm power levels. The noise of a SC is known to be wavelength dependent, so the noise of the OCT system will also depend on wavelength or pixel number. Figure 2a–c shows a representative example at 1432 nm, for the variance of the three sources as a function of mean counts (averaged over 1024 measurements). The circles are the measured values and the continuous line is the fit for the total variance, ${\sigma }_{tot}^{2}\,=\,{\sigma }_{r\,+\,d}^{2}+{\sigma }_{shot}^{2}\,+\,{\sigma }_{ex}^{2}$. Here ${\sigma }_{r\,+\,d}^{2}$ is the read out and dark noise from the spectrometer, which is independent of input power. ${\sigma }_{shot}^{2}$ is the shot-noise, which obeys Poisson statistics and scales linearly with the power incident on the spectrometer. ${\sigma }_{ex}^{2}$ is the excess photon noise, in this case due to the pulse-to-pulse relative intensity noise (RIN) fluctuations of the SC source. ${\sigma }_{ex}^{2}$ scales with the square of the incident power.

The different dependencies of the variance on the power allows for separation of the three noise sources. The variance should first of all go towards the same spectrometer-determined value ${\sigma }_{r\,+\,d}^{2}$ for all sources for very low mean counts, which is also confirmed by the experiments. By doing a least-square fit of the measured variance to a linear combination of the three individual noise terms, we have quantified their relative strength and plotted them as dashed lines in Fig. 2a–c. From Fig. 2a, b we see that for the 80 and 320 MHz SuperK extreme sources, the variance of the noise is dominated by excess noise from the SC source for readings that exceed 250 counts, corresponding to just 6% of the 12 bit dynamic range.

Our results clearly show that the high RIN of the 80 and 320 MHz SuperK extreme sources does not allow the OCT system to be operated in the shot-noise limited region, as otherwise reported in ref. ^29^. Here it is important to realize that when the total variance goes from being limited by the read out and dark noise of the detector at low counts (${\sigma }_{r\,+\,d}^{2}$ with slope 0 in Fig. 2a–c) to being limited by the excess noise or RIN of the source at high counts (${\sigma }_{ex}^{2}$ with slope 2 in Fig. 2a–c), it will always transition through a certain local region of intermediate count values with slope 1. This can wrongly be interpreted as being shot-noise limited if one only locally fits a straight line with slope 1 to the experiments and not surprisingly finds that it fits for a narrow range of count values. One would for example wrongly predict even the 80 MHz SuperK extreme source to provide shot-noise limited operation by doing so, which it clearly does not. The correct approach is to fit the entire variance versus counts data set to a linear combination of the three noise terms, as we do here, to clearly see their relative strength and be able to determine whether the total variance is shot-noise limited or not.

In sharp contrast to what was found for the SuperK extreme sources, the excess noise of the ANDi SC source, shown in Fig. 2c, never dominates within the dynamic range of the spectrometer, and for readings over 1000 counts the recorded variance remains shot-noise limited. Figure 2d, e show two more representative examples, at 1326 and 1394 nm, comparing the variance of each source. Here, it can be seen that by using the ANDi SC source for a fixed number of counts of 1100, the noise characteristics of the OCT system is improved by 12 dB when compared to the 80 MHz SuperK extreme source and by 10 dB when compared to the 320 MHz SuperK extreme source. We note that the dependency of the noise on the number of counts is qualitatively the same at all wavelength within the bandwidth of the source, for all the three sources.

For a fair comparison of the OCT performance of the three sources we always operate the OCT system at a number of counts that gives the optimum sensitivity performance. For the two SuperK extreme sources this is when the read out and dark noise level equals the excess noise level^7,30^, which is at 250 mean counts at 1432 nm (see Fig. 2a, b). For the ANDi SC source this point is below the shot-noise limit and thus cannot be reached. The optimum point of operation is then at the highest number of counts that does not saturate the detector. We therefore chose to operate the ANDi SC source at a fixed number of counts of 1000 at 1432 nm.

To compare the influence of the SC noise on the quality of the A-scans, Fig. 2g shows the variance of the A-scans from the reference arm alone for the three sources, all processed with dark noise subtraction, normalization to the mean of the reference, and spectral apodization using the window shown in Fig. 2f. It can be seen that the ANDi SC source provides a significant improvement in A-scan variance for all optical path differences (OPDs) of about 13 dB compared to the 80 MHz SuperK extreme source and 9 dB compared with the 320 MHz SuperK extreme source. This clearly demonstrates that the lower noise level of the ANDi SC source will result in a much better (darker) background in SD-OCT imaging compared to what can be obtained with even the state-of-the-art commercial SuperK extreme sources.

The performance of an OCT system is most commonly characterized by its sensitivity, which is directly related to the noise. As we show in the materials and methods section and related Fig. 6f–h, the low-noise of the ANDi SC source provides a remarkable 12 dB increase in sensitivity compared to the 80 MHz SuperK extreme source, which is the direct comparison in terms of repetition rate, and of 7 dB with respect to the 320 MHz SuperK extreme source. Our noise and sensitivity characterization of the three SC sources therefore shows that the ANDi SC source provides shot-noise limited SD-OCT imaging with the lowest background noise and highest sensitivity. This is the first demonstration of shot-noise limited SC-based SD-OCT operation and, as we shall see in the next section, it translates into a significant improvement in image quality.

### Images

The OCT setup was first characterized by its axial resolution using the three sources. The wavelength region 1280–1478 nm used in the OCT measurements was found to produce an axial resolution of 5.9 μm. The lateral resolution was 6 μm^11^. Each of the samples were imaged with the same settings in the OCT system. Identical gray-scaling was ensured for image comparisons. All images are displayed on a logarithmic scale.

**Fig. 3:**
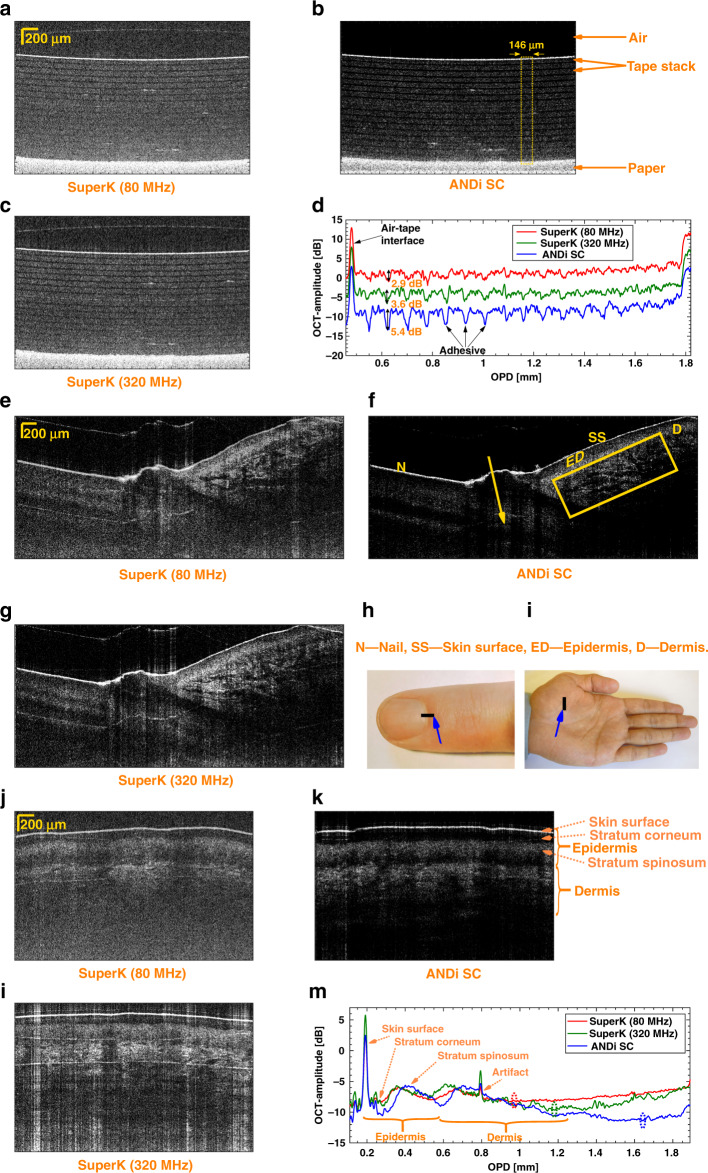
OCT imaging of tape layers and a finger. *Single B-scans of tape layers* (2 × 3 mm), obtained using the 80 MHz SuperK extreme (**a**), ANDi SC (**b**), and 320 MHz SuperK extreme (**c**) sources. Averaged A-scans within the 146 μm wide dotted box in (**b**) for the three sources offset by 5 dB (**d**). The average is taken over 49 A-scans equally spaced within the dotted box. *Single B-scans of a finger where the nail begins* (2 × 4.5 mm) [see photo (**h**)], obtained using the 80 MHz SuperK extreme (**e**), ANDi SC (**f**), and 320 MHz SuperK extreme (**g**) sources. *Single B-scans of the rough skin on a hand palm* (2 × 3 mm) [see photo (**i**)] obtained using the 80 MHz SuperK extreme (**j**), ANDi SC (**k**), and 320 MHz SuperK extreme (**l**) sources. Averaged A-scan (averaged over entire B-scan, corresponding to 1024 A-scans) of the rough palm skin for the three sources (**m**). Dashed ellipses mark the penetration depth

In order to easily compare the image quality from each source, we first imaged a stack of 17 layers of tape, each layer of tape consisting of a highly scattering layer of plastic and a thin less scattering layer of adhesive. The single B-scans with an area of 2 (axial) × 3 (lateral) mm is shown in Fig. 3a, c for the 80 MHz SuperK extreme, ANDi SC, and 320 MHz SuperK extreme sources, respectively. The ANDi SC source gives the largest signal contrast of the reflections from the plastic to adhesive interfaces as expected. Both the ANDi SC and the 320 MHz SuperK extreme sources enables the OCT system to see through all layers and see the paper substrate, while the 80 MHz SuperK extreme source does not allow to clearly distinguish the bottom interfaces. Figure 3d shows averaged A-scans within the 146 μm wide dotted box in Fig. 3b. The average is taken over 49 A-scans equally spaced within the dotted box. The plastic is scattering much more than the adhesive, and thus the broad maxima in the A-scans represent the plastic, while the narrow dips represent the adhesive. The amplitude of the A-scans, for the individual sources, are offset by 5 dB for clarity. We see from the averaged A-scan using the ANDi SC source that the (adhesive) dips are clear until the end of the sample and gives a 5.4 dB contrast in the second adhesive layer, as shown in Fig. 3d. In contrast the 80 and 320 MHz SuperK extreme sources only give a contrast of 2.9 and 3.6 dB, respectively. It is important to note that in order to assess the influence of the pulse-to-pulse source noise in images, the ANDi SC source should be compared with the 80 MHz SuperK extreme source, as they have similar repetition rates and therefore the same degree of averaging. By demonstrating that the ANDi SC provides a better signal contrast of the images than what is obtained using the 320 MHz SuperK extreme, we show that even the current state-of-the-art in commercial SC sources specifically designed for OCT, provides significantly poorer image qualities.

To study the difference in the images when the OCT system is used to image laterally inhomogeneous biological samples, we first imaged the skin of a healthy volunteer. Images of a 2 × 4.5 mm section of an index finger nail fold and a 2 × 3 mm section of a palm are shown in Fig. 3e–g, j–l, respectively. Using a marker it was assured that the nail-fold images were of the same tissue segment, so that a quantitative comparison is justified. By comparing the nail-fold images obtained when using the ANDi SC source and the 80 MHz SuperK extreme source, it can be seen that the region around and below the nail, as it goes into the finger (arrows), is clearly visible in the image with the ANDi SC source, but not visible in the image obtained by using the 80 MHz SuperK extreme source and only barely visible with the 320 MHz SuperK extreme source. In addition, the tissue segment of the nail-fold capillary network in the right side of the image (rectangle), is seen with a higher contrast and larger imaging depth for the ANDi SC source.

To quantify the increased penetration, we imaged the hand palm, which has a more regular surface than the nail fold, see Fig. 3j–l. For the palm the imaged area is only approximately the same and while the images with the 80 MHz SuperK extreme and the ANDi source are taken shortly after one another, the image with the 320 MHz SuperK extreme is taken several days later. This means that the images can only be qualitatively compared. As with the nail fold, the ANDi SC source is seen to provide a darker background, higher contrast, and improved penetration. Figure 3m shows A-scans for each source, averaged across the entire B-scan (1024 A-scans). The curves are shifted horizontally and vertically such that the major peak from the air–skin interface overlap, allowing a relative comparison of the signals from within the skin. We see that the epidermis and dermis signals (indicated local broad maxima) are of similar strength with all three sources, but that the dark part in the stratum corneum is darker when the ANDi SC is used. Also, the 80 MHz and 320 MHz SuperK extreme curves hit the noise floor at a penetration depth of 0.78 and 0.96 mm below the surface, respectively, whereas the ANDi SC first reaches the noise floor at 1.45 mm below the surface. The measured penetration depths (marked by a dashed ellipse on the curves) demonstrate that the ANDi SC source provides an increased penetration depth of about 0.5 mm even compared to the 320 MHz SuperK extreme source.

**Fig. 4:**
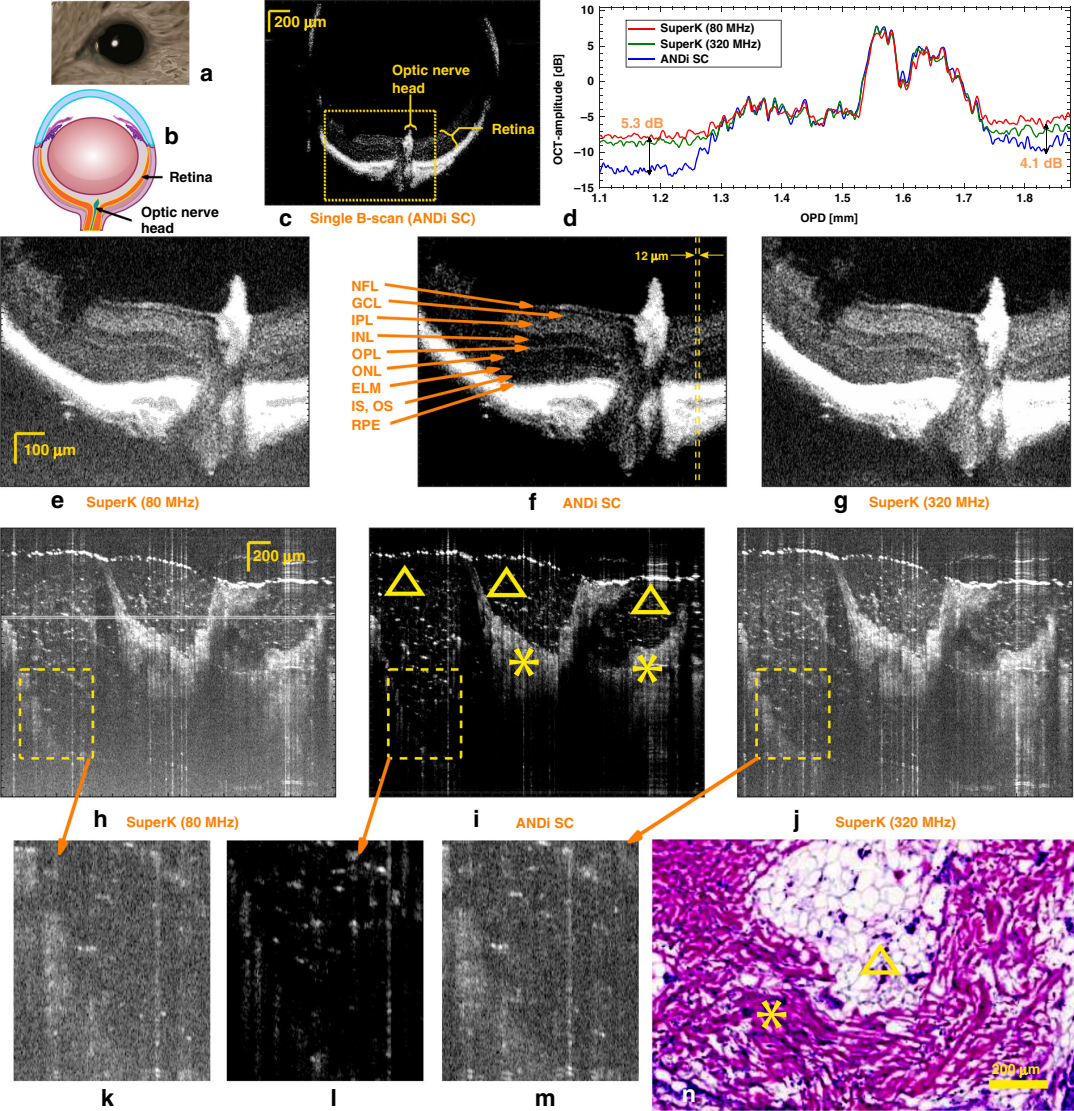
OCT imaging of rat eye and human fat tissue. *Ex vivo rat-eye imaging:* Artistic image of a mouse eye (**a**) with a cross-sectional schematic shown in (**b**). Single B-scan OCT image in depth of a 1.95 × 2.62 mm section of a mouse retina obtained using the ANDi SC source (**c**). Averaged A-scans (averaged over 4 equally spaced A-scans) from the marked region in (**f**) for the three sources (**d**). Zooms of the retina and optic nerve in the area marked by a yellow box in (**c**) using the 80 MHz SuperK extreme (**e**), ANDi SC (**f**), and 320 MHz SuperK extreme (**g**) sources (all averaged over 9 B-scans). NFL nerve fiber layer, GCL ganglion cell layer, IPL inner plexiform layer, OPL outer plexiform layer, ONL outer nuclear layer, ELM external limiting membrane, IS, OS inner segments, outer segments of the photoreceptors, and RPE retinal pigment epithelium. *Ex vivo imaging of human fat tissue*: 1.9 × 3 mm human skin biopsy averaged over nine single B-scans, obtained using the 80 MHz SuperK extreme (**h**), ANDi SC (**i**), and 320 MHz SuperK extreme (**j**) sources. Histology of fat tissue (**n**). Triangles and stars mark tissue types of cutaneous fat tissue and connective tissue of reticular dermis, respectively

To demonstrate the image quality improvement within ophthalmology, we imaged a mouse retina sealed in epon resine^31^, with each of the three sources. A single B-scan image of the full mouse retina, obtained using the ANDi SC source, is shown in Fig. 4c. In Fig. 4e–g we zoom on the lower part of the retina around the optic nerve, corresponding to the region in Fig. 4c marked with a dashed box, and show averaged B-scans (averaged over 9 B-scans) to better highlight individual features, such as layers of different cell types and the optic nerve head. The various cell layers are labeled in Fig. 4f. Since the retina is thin and transparent, all the three sources were able to penetrate the entire retina, but the ANDi SC source provides a much better contrast. The improvement in the contrast is up to 5.3 dB, as can be seen quantitatively from the averaged A-scans for the three sources shown in Fig. 4d, obtained by averaging across the 12 μm region within the two dashed lines in Fig. 4f.

To further demonstrate the sensitivity enhancement obtained by using the ANDi SC source, we imaged a different type of tissue being that of cutaneous fat tissue found in the dermis of the skin. A biopsy was extracted according to the procedure of Mohs surgery. After defrosting the biopsy it was imaged with the three different sources. The result (averaged over nine individual B-scans) are seen in Fig. 4h–j. Due to the already demonstrated better contrast, the ANDi SC source provides a significantly improved information content, which is especially observed in the deeper parts of the tissue. From the zooms presented in Fig. 4k–m we see for example that the scattering points from the hexagonal-like structures of the cutaneous fat tissue (marked by triangles in Fig. 4h–j and the histology image in Fig. 4n), are much more clearly recognized with the ANDi SC source.

In general, the quality of the images obtained with the 320 MHz SuperK extreme source is better than the ones obtained using the 80 MHz SuperK extreme source, as expected due to the higher repetition rate and thereby increased averaging. However, even with 3.6 times lower repetition rate the ANDi SC source performs better than the 320 MHz SuperK extreme source because of its extraordinary low-noise properties.

## Discussion

### Achievable sensitivity

**Fig. 5:**
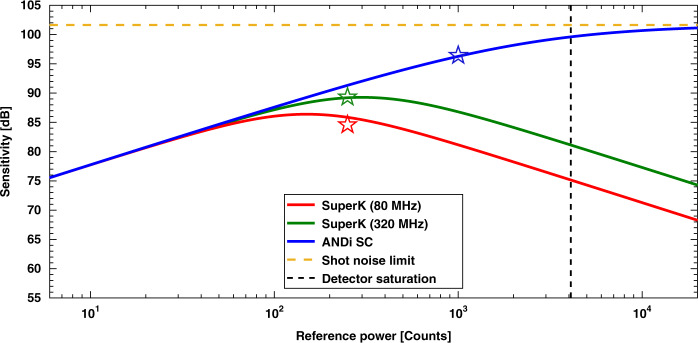
OCT sensitivity. Theoretical sensitivity as a function of reference power for the 80  MHz SuperK extreme, 320  MHz SuperK extreme, and ANDi SC sources. Stars represent the experimentally obtained sensitivities presented in the materials and methods section. Model parameters are found from experimental system characterization and are given in detail also in the materials and methods section. Calculations are based on refs. ^6,16^

In order to assess the sensitivity values produced by the three source configurations for arbitrary values of the reference power, we have modeled the sensitivity theoretically under conditions of the experiments. The theoretical description of the OCT sensitivity is well-known and is commonly in short form expressed as the signal-to-noise ratio (SNR) for a perfect reflector^6^: Sensitivity $\,=\,SN{R}_{max}\,=\,{\tilde{S}}_{max}^{2}/\left(\right.{\tilde{\sigma }}_{r\,+\,d}^{2}\,+\,{\tilde{\sigma }}_{shot}^{2}\,+\,{\tilde{\sigma }}_{ex}^{2}\left)\right.$ with all components now evaluated in the OPD domain, signified by a tilde. Here ${\tilde{S}}_{max}$ is the maximum of the detected point spread function of the reflector and the sigmas are the respective noise contributions from spectrometer, shot-noise and excess noise from the SC source. The traditional representation of the RIN (and therefore ${\tilde{\sigma }}_{ex}^{2}$) as being that for a thermal source has been shown to overshoot the RIN of a SC source and we thus apply a more precise measurement-based model^16^. With this, we present the theoretically calculated sensitivities for the three sources in Fig. 5 as a function of power returned from the reference arm. The sensitivities for the experimentally used optimum (for our systems) reference power settings are marked by stars.

From Fig. 5, we find the expected behavior of the sensitivity for the three different sources. The 80 and 320 MHz SuperK extreme sources must be operated at the reference power, at which the total noise becomes dominated by the RIN of the source, which limits the maximum sensitivity. In contrast, when using the ANDi SC source, the reference power can be increased to a level close to detector saturation in order to approach the shot-noise limit of maximum sensitivity, which is 102 dB for our system (yellow dashed line in Fig. 5). Due to the high read out and dark noise of the InGaAs (indium gallium arsenide) detectors, the sensitivity value of our system is 96 dB, at the reference power level of 1000 counts. This is 7 dB above the maximum achievable sensitivity when using the 320 MHz SuperK extreme source, but still 6 dB below the maximum shot-noise limit.

The sensitivity level of an OCT system depends on several factors, such as power, integration time, the quality of the detector, and the optical loss from the sample to the detector. A detector with a higher saturation threshold would allow to operate closer to the shot-noise limit and thereby improve the sensitivity. Furthermore, the loss of a broadband fiber-based SD-OCT system as ours is rather high, mainly due to losses in the coupling to and from the fibers and the fiber coupler. A characterization showed that only a 6% fraction of the power returned from the sample reached the spectrometer. A reduction of the loss would significantly improve the obtainable shot-noise limited sensitivity. A realistic improvement of the fraction of returned power of, for example, a factor of two would intrinsically lift the achievable shot-noise limit to 105 dB and the sensitivity in our experimental setting to 99 dB.

With this in mind it is interesting to discuss the work from 2016 by Yuan et al. in which they reported the current record sensitivity of 107 dB for SC-based SD-OCT, when using the commercial 320 MHz SuperK extreme source from NKT Photonics, with similar sample power and spectrometer integration time and depth resolution as we use here^29^. The sensitivity result of theirs is thus directly comparable to those obtained with the 320 MHz SuperK extreme source presented in this paper. The main difference is that in ref. ^29^ the OCT system was operated at 800 nm with a silicon based spectrometer, so both the detector and the optical system is different from our system. However, assuming that the relative improvement of 7 dB when going from 320 MHz SuperK extreme to ANDi SC still holds, this would translate our ANDi SC result to a record 114 dB sensitivity using their 800 nm SC-based OCT system.

The record sensitivity of 96 dB achieved here with the ANDi SC-based 1300 nm OCT system and potential 114 dB achievable at 800 nm, is competing with the leading OCT technology in terms of sensitivity, which is SS-OCT. SS-OCT is a technology that in most cases musters a 105–110 dB OCT sensitivity but is limited to a spectral bandwidth of only about 150 nm in, e.g., the 1300 nm wavelength range^2,32,33^, which means that it has a limited axial resolution of about 7 μm. Our results predict a future ANDi SC-based SD-OCT system, which has the ultra-high axial resolution of SD-OCT systems and the ultra-high sensitivity of SS-OCT systems.

### Conclusion

SC research has unlocked an unprecedented axial resolution in OCT. The gist of the technology being that the axial resolution is fundamentally decoupled from the choice of focus of the scanning beam and instead determined by the spectral bandwidth of the light source.

In this work, we have shown that an ANDi SC source, in which the SC is generated using an ANDi fiber pumped by a short fs pulse, can have so low-noise that it enables excess noise-free SD-OCT. Using an SD-OCT system, for a spectrum with a center wavelength of 1370 nm, we have compared the noise properties of the ANDi SC source with two commercially available SC sources carrying the disreputable high RIN. Using the ANDi SC source, we have for the first time demonstrated that SD-OCT can now be operated in the shot-noise limited detection regime. With the improved noise characteristics, we find that an OCT sensitivity gain of 12 dB is inherently won, while maintaining an axial resolution of 5.9 μm and with only a slight increase of repetition rate from 80 to 90 MHz, as presented in Fig. 2d–g.

The value of this improvement is first of all manifested in the increased contrast of the retinal layers of the eye against the background, in this work represented by an ex vivo mouse retinal sample depicted in Fig. 4a–c. The lower background noise enables faster imaging without significant loss of image quality. With OCT known for revolutionizing the ophthalmological diagnosis, the performance of delineating the retinal cell layers to a high precision is decisive and this demands both best possible performance in axial resolution and sensitivity, when tracking individual cell layers.

In dermatology, OCT has proved to increase the diagnostic performance of skin cancer, achieving an accuracy for basal cell carcinoma of ~60% and in melanoma of ~40%^34^. Finding the transition between malignant and benign tissue and dismissing cancer tissue emitators in OCT images is however still very challenging. By imaging a nail fold, a hand palm and an ex vivo subcutaneous fat tissue, we have documented a substantial improvement in cell structure transitions as well as increased penetration depth, the latter being ever important in the pursue of recognizing and removing the full extension of malignant tissue.

In general, the noise properties and the images obtained by using the 80 and 320 MHz SuperK extreme sources show that having a high repetition rate improves the details in the image, when the sources have high pulse-to-pulse fluctuations. The ANDi fiber-based low-noise source, on the other hand, does not rely on pulse averaging to keep the noise low and is therefore a prime candidate to take advantage of the ever-increasing spectrometer speeds without sacrificing image quality. Importantly, recently an ANDi spectrum covering 670–1390 nm was generated using a compact externally compressed 1030 nm fs laser^35^. This means that the low-noise ANDi SC source technology demonstrated here can soon become available also for SD-OCT around 800 nm, commonly used in opthalmology.

## Materials and methods

### Experimental setup

**Fig. 6:**
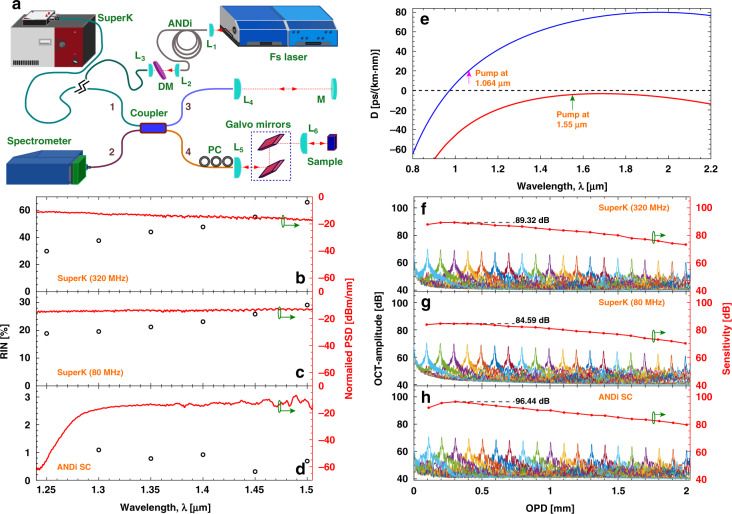
OCT system and SC source characterization. Schematic of the OCT setup (**a**) [L_*i*_ lens, M mirror, DM dichroic mirror, and PC polarization controller]. Normalized power spectral density (PSD) profiles measured using an OSA (red curves, right axis) and RIN profiles (black circles, left axis) for the 320 MHz SuperK extreme (**b**), the 80 MHz SuperK extreme (**c**), and the ANDi based low-noise SC source (**d**). Calculated dispersion profile for the nonlinear fiber used in the SuperK extreme (blue) and the measured dispersion of the GeO_2_ doped silica ANDi fiber (red) (**e**). Mean OCT-amplitude (over 1024 measurements) of a sample mirror at 20 different axial positions on the left axis, and the sensitivity on the right, for the SuperK extreme at 320 MHz (**f**) and 80 MHz (**g**), and the ANDi based low-noise SC source (**h**)

The SD-OCT setup, shown in Fig. 6a, is a Michelson interferometer with an ultra-broadband 50/50 coupler (Thorlabs: TW1300R5A2) splitting the light into a reference arm (arm labeled “3”) and a sample arm (arm labeled “4”). The sample arm contains a two-axis galvo scanner (Thorlabs: GVS002) after which the light is focussed onto the sample using a lens. The spectrometer in the arm labeled “2” has a detection range from 1074 to 1478 nm with a resolution of 0.2 nm (Wasatch Photonics: C-1070-1470-GL2KL). The spectrometer has a 12 bit quantization for the amplitude and 2048 pixels. In the source arm “1” three different sources were used. For the low-noise ANDi SC source, a 125 fs full width at half maximum pump at 1.55 μm (Toptica: Femto fiber) operating at 90 MHz repetition rate was coupled into 10 m of GeO_2_ doped silica fiber (OFS Denmark) using an aspheric lens. The fiber has an ANDi profile. The dispersion of the fiber was measured from 0.86 to 2.38 μm using white light interferometry^36^ and is plotted in Fig. 6e (red). The SC generated in the ANDi fiber spans a bandwidth of 1280–1910 nm at the −30 dB level.

The light out of the ANDi fiber was collimated using an aspheric lens and a dichroic mirror (Thorlabs: DMSP1500), labeled “DM”, was used to cut off light above 1500 nm. The light was then free space coupled into arm “1” of the interferometer using an aspheric lens. The other two sources used were a SuperK extreme operating at a repetition rate of 80 MHz (NKT Photonics: SuperK EXTREME EXW-12) and a SuperK extreme operating at a repetition rate of 320 MHz (NKT Photonics: SuperK EXTREME EXR-9 OCT) developed specifically for OCT. The calculated dispersion of the nonlinear fiber in these sources is plotted in Fig. 6e (blue). The SC was spectrally filtered using a long pass filter at 1.0 μm (Thorlabs: FELH1000) and a short pass filter at 1.5 μm (Thorlabs: DMSP1500) and fiber coupled to arm “1” of the interferometer. For direct comparison of the three sources, it was ensured that in all OCT measurements (i.e., all results shown in all the figures), the average power in the sample arm was fixed at ~3.5 mW. The integration time of the spectrometer for all of the measurements was 9.2 μs and the spectral line scan rate was 76 kHz.

### Spectra and RIN measurement

The spectra used for the OCT measurements are shown in Fig. 6b–d (red, right axis). When a fs pulse is launched into a fiber with a weak ANDi profile the spectral broadening is dominated by SPM and OWB and these broadening processes can lead to a coherent spectrum. For the SC from the SuperK extreme sources the nonlinear fiber has a zero dispersion wavelength around 0.98 μm, as shown in Fig. 6e (blue), and a ps pump centered at 1.064 μm is used. The spectral broadening is dominated by modulation instability, which generates a distributed spectrum of solitons in the anomalous dispersion regime. The solitons then interact and collide while red-shifting due to the Raman-induced soliton self-frequency shift. In this case, the noise property of the generated spectra is dominated by the amplification of quantum noise and subsequent phase- and amplitude-dependent soliton collisions, which make the spectra temporally incoherent. Even though the spectra measured by the optical spectrum analyser (Yokogawa: AQ6317B) show a flat profile in Fig. 6b, c, there is, in fact considerable spectral fluctuations from pulse-to-pulse. In order to quantity these fluctuations, we performed spectrally resolved pulse-to-pulse RIN measurements^37,38^. The spectrally resolved RIN measurements were performed by using several 10 nm bandpass filters in intervals of 50 nm. The bandpass filtered SC was coupled into a 50 μm core diameter fiber. This was then fiber-coupled into a 5 GHz bandwidth InGaAs detector with an active area diameter of 80 μm (Thorlabs: DET08CFC). The pulse train out of the photodiode was recorded with a fast oscilloscope (Teledyne: LeCroy-HDO9404, 4 GHz bandwidth, 40 GS/s). The linear region of the detector was initially determined by using the bandpass filtered spectrum from the pump. During the measurement of the RIN of the SC, it was ensured that the detector was being operated in this linear regime by suitably adjusting the power into the detector. The peaks of the measured trace, which are proportional to the pulse energy, were extracted and used to find the RIN = *σ*/*μ*, where *σ* is the standard deviation, and *μ* is the mean. For the ANDi fiber-based SC source 45,135 pulses were used in the measurement of each of the RIN value and the measured RIN values are shown in Fig. 6d (left axis). The excellent noise properties of the ANDi fiber-based SC source is confirmed by the low RIN values, which are below 1.1% in the entire wavelength range. For the 80 MHz SuperK extreme source, 249,078 pulses were used in the measurement of each of the RIN value and the measured RIN values are shown in Fig. 6c (left axis). The RIN is 18.9% at 1.25 μm and increases to 29.2% at 1.5 μm. Similarly, for the 320 MHz SuperK extreme source, 997,288 pulses were used in the measurement of each of the RIN value and the measured RIN values are shown in Fig. 6b (left axis). The RIN is 29.9% at 1.25 μm and increases to 66% at 1.5 μm. The 320 MHz SuperK extreme source has higher pulse-to-pule RIN than the one operating at 80 MHz. However, in the OCT setup, the spectrometer records lower noise for the 320 MHz SuperK extreme source (see Fig. 2a, b, d, e) as the spectrometer averages over time and the 320 MHz SuperK extreme source has four times more pulses in a given integration time than the 80 MHz SuperK extreme source. These measurements show that the ANDi SC source has very low pulse-to-pulse fluctuation, more than an order of magnitude lower than the commercially available SC sources.

### Characterization of the OCT setup

The axial resolution of the OCT system was determined to be 5.9 μm, averaged over 20 OPDs of the mirror from 0.1 mm (from zero OPD) to 2 mm (from zero OPD) in steps of 0.1 mm. No significant broadening was observed at larger OPD (when the ANDi SC was used, e.g., the axial resolution was 5.77 μm at 0.5 mm, which increased to 5.96 μm at 1.8 mm). The lateral resolution was measured to be 6 μm^11^. In OCT, the sensitivity is defined as the minimum sample reflectivity, which gives a SNR of unity. The sensitivity is calculated as $SN{R}_{max}\,=\,{\tilde{S}}_{max}^{2}/{\tilde{\sigma }}_{tot}^{2}$^39^. After the reference power was adjusted such that the measured photon-counts alone from the reference arm was set at the optimum point; that being ~250 counts for the SuperK extreme sources, a mirror was placed in the sample arm. The reflection of the full power would saturate the spectrometer, so the sample mirror was slightly misaligned to attenuate the power collected by the OCT system below the saturation limit. This attenuation was added to the signal/noise fraction to obtain the sensitivity. The sensitivity roll-off represents the signal degradation with the OPD due to both finite pixel size and spectral resolution in the spectrometer. The sensitivity roll-off of the OCT system was measured to be 9.5 dB/mm when using the 320 MHz SuperK extreme source, 8.0 dB/mm when using the 80 MHz SuperK extreme source, and 8.8 dB/mm when the ANDi SC source was used.

### Human volunteers and image processing

In vivo images of human skin was carried out on two volunteers and was part of a larger study approved by the local ethics committees of Bispebjerg Hospital, Denmark, (journal no. H-19036900). Written and orally informed consent was obtained before enrollment in the study, which was conducted in accordance with the Declaration of Helsinki.

During the imaging, the sample was positioned at a negative OPD such that the deeper layers were closer to OPD = 0 mm. This was done in order to have the sensitivity roll-off work against the scattering of the sample, such that the deeper layers with weak signal were in the lower OPD region with high sensitivity. All the images are plotted on a logarithmic scale. A Hamming window was used in the wavelength range 1280–1478 nm and zeros was used at 1074–1280 nm. The value provided for the axial depth for each of the images is the corresponding OPD.

### Calculation of theoretical sensitivity curves

The values used to obtain the theoretical sensitivity curves, plotted in Fig. 5, are based on the equation for sensitivity denoted as Σ_*FDOCT*_ in ref. ^6^ with the RIN extension of^16^, and are provided in the following: The fraction of the total power from the sample arm exiting the interferometer is *γ*_*s*_ = 0.063 (measured), the fraction of the total power from the reference arm exiting the interferometer is *γ*_*r*_ = 0.133 (measured), the spectrometer efficiency (losses in spectrometer before hitting the detector) is *ρ* = 0.5 (from vendor), the quantum efficiency of the detector is *η* = 0.7 (from vendor), the exposure time of the detector is *τ* = 9.2 μs, the center wavelength is *λ*_*c*_ = 1370 nm, the polarization degree is assumed to be zero, the power at the center wavelength (measured sample and reference power in total) is *P*_0_ = 10.4 mW, the spectrometer gain factor is Δ*e* = 270 electrons/count, and the read out and dark noise is *σ*_*r* + *d*_ = 810 electrons, the latter two specified by the vendor.

## Supplementary information

Supplementary information for Shot-noise limited, supercontinuum-based optical coherence tomography

## Data Availability

All data needed to evaluate the conclusions in the paper are present in the paper. Additional data related to this paper may be requested from the authors.
