# Supplementary material for: Shot-noise limited, supercontinuum-based optical coherence tomography
Source: Light Sci Appl. 2021 Jun 28;10:133. doi: 10.1038/s41377-021-00574-x (PMC8239030; doi:10.1038/s41377-021-00574-x)
Supplement: Supplementary file 1 — Supplementary information for Shot-noise limited, supercontinuum-based optical coherence tomography [file 41377_2021_574_MOESM1_ESM.pdf]

Supplementary information for

Shot-noise limited, supercontinuum based optical coherence tomography

Shreesha Rao D. S.[1], Mikkel Jensen[1], Lars Grüner-Nielsen[1], Jesper Toft Olsen[2], Peter Heiduschka[3], Björn Kemper[4], Jürgen Schnekenburger[4], Martin Glud[5], Mette Mogensen[5], Niels Møller Israelsen[1], and Ole Bang[1,2,*]

[1]*DTU Fotonik, Dept. of Photonics Engineering, Technical University of Denmark, Ørsteds Plads, Kongens Lyngby, 2800, Denmark.*
[2]*NKT Photonics A/S, Blokken 84, 3460 Birkerød, Denmark.*
[3]*Department of Ophthalmology, University of Münster Medical Centre, Domagkstr. 15, Münster, D-48149, Germany.*
[4]*Biomedical Technology Center of the Medical Faculty, University of Münster, Mendelstr. 17, Münster, D-48149, Germany.*
[5]*Department of Dermatology, Bisbebjerg Hospital, University of Copenhagen, Bispebjerg Bakke 23, Copenhagen NV, 2400, Denmark.*
[*]*oban@fotonik.dtu.dk*

The PDF file includes:
Supplementary Text
Figs. S1 to S3
References

Low-noise supercontinuum generation

The full spectrum of the ANDi fiber based low-noise supercontinuum (SC) source spanning from 1.28 to 1.91 μm at the -30 dB level is shown in Fig. S1 (solid). The length of the ANDi fiber used is 10 m. In order to numerically simulate the SC, the single-polarization generalized nonlinear Schrödinger equation (GNLSE) was solved in the interaction picture. The time-domain GNLSE [1] with electric field envelope was transformed to the frequency-domain interaction picture equation [2, 3]. For the germania doped silica based ANDi fiber, the experimentally found dispersion, and effective area (A_{eff}) of the fiber at the pump provided by the manufacturer were used in the simulations. A_{eff} used in simulation was 11.25 μm^2. As the autocorrelation measurement of the pump pulse

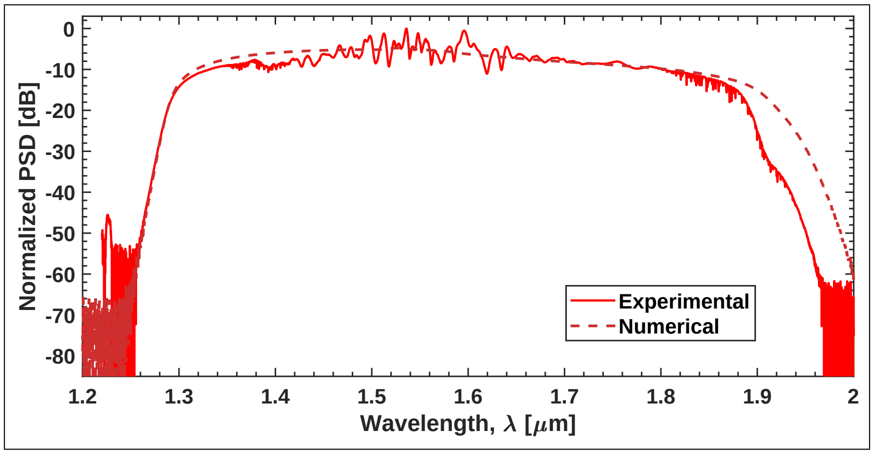

Fig. S1: Numerical simulation (dashed) and experimentally measured spectra (solid) out of the fiber for $P_0 = 9$ kW coupled into 10 m length of the fiber.

fitted well with a secant hyperbolic pulse with $T_{FWHM} = 125$ fs, this was used as the input pulse in the simulations. The numerically found spectrum with a peak power (P_0) of 9 kW for 10 m length of the fiber is overlaid with the experimentally measured spectrum in Fig. S1 (dashed line). A good correspondence is observed.

The power spectral density (PSD) evolution is shown in Fig. S2(A). The numerical simulation of the temporal evolution of the pulse along the length of the fiber is shown in Fig. S2(B), plotted in logarithmic scale. As is typical for the temporal evolution in an ANDi fiber, the output is made of a single smooth pulse. We observe that the spectral

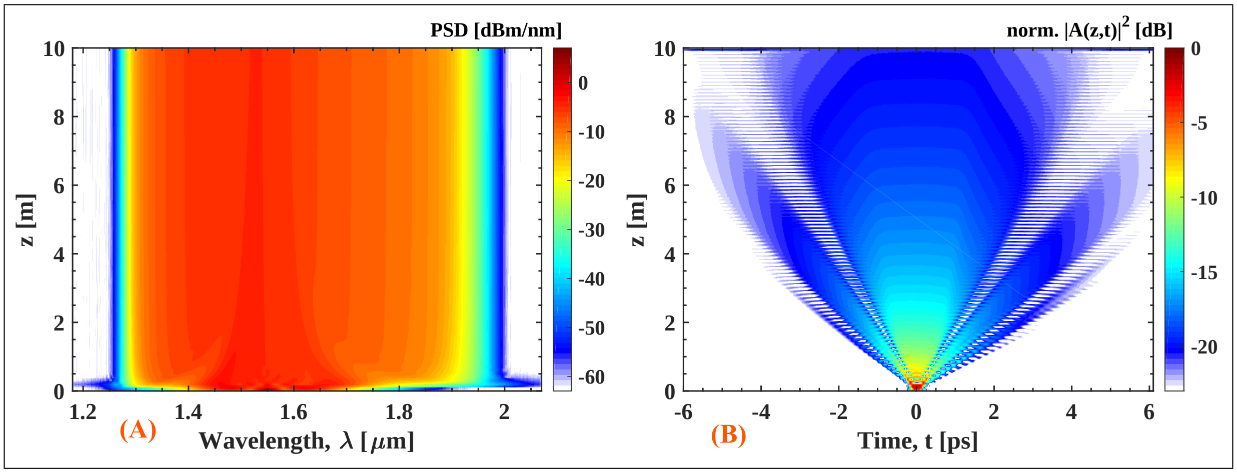

Fig. S2: Numerical simulations with $T_{FWHM} = 125$ fs, $P_0 = 9$ kW, and $z = 10$ m. **(A)** PSD evolution along the length of the fiber. **(B)** Evolution of the pulse in time along the length of the fiber, plotted in logarithmic scale.

broadening primarily takes place within the first 0.3 m, and further propagation up to 10 m just flattens the spectrum, as seen in Fig. S2(A). As the spectrum coherently broadens through self-phase modulation (SPM) and optical wave

breaking (OWB), a spectrum with extremely low pulse-to-pulse fluctuation can be obtained. The initial broadening from SPM and the later broadening from OWB can be clearly seen from a zoom on the evolution in the first 1 m of propagation shown in Fig. S3(A). The numerical simulation of the temporal evolution of the pulse along the first 1 m length of the fiber is shown in Fig. S3(B), normalized and plotted in linear scale.

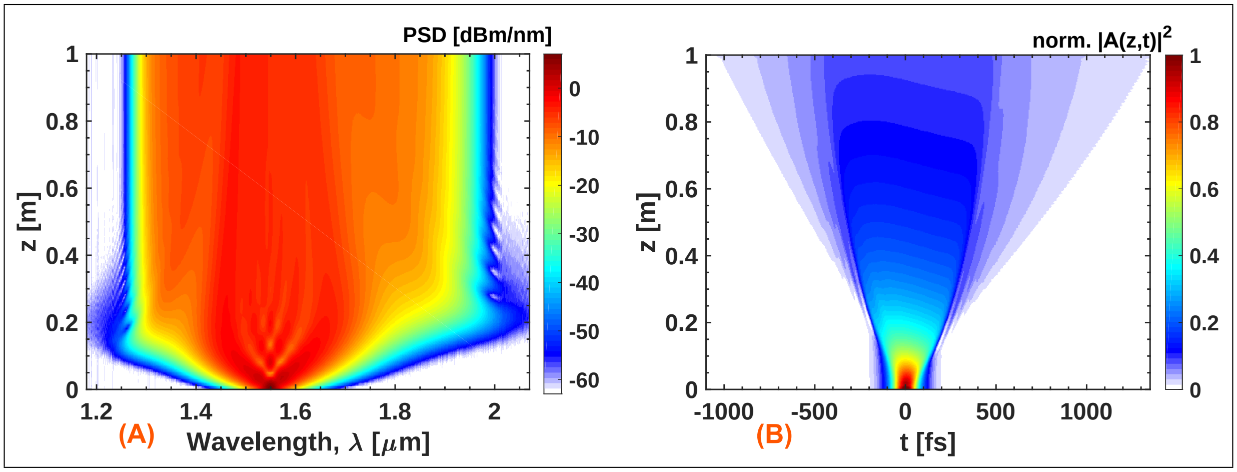

Fig. S3: Numerical simulations with $T_{FWHM} = 125$ fs, $P_0 = 9$ kW, and $z = 1$ m. **(A)** PSD evolution along the first 1 m length of the fiber. **(B)** Evolution of the pulse in time along the first 1 m length of the fiber, normalized and plotted in linear scale.

REFERENCES

[1] G. P. Agrawal. *Nonlinear Fiber Optics*. 5th ed. Academic Press, 2012.

[2] J. Hult. "A fourth-order Runge–Kutta in the interaction picture method for simulating supercontinuum generation in optical fibers". *Journal of Lightwave Technology* 25.12 (Dec. 2007), pp. 3770–3775.

[3] J. M. Dudley and J. R. Taylor. *Supercontinuum generation in optical fibers*. Cambridge University Press, 2010.
